# A Brief Digital Screening and Intervention Tool for Parental and Adolescent Tobacco and Electronic Cigarette Use in Pediatric Medical Care in Canada: Protocol for a Pilot Randomized Controlled Trial

**DOI:** 10.2196/47978

**Published:** 2023-11-30

**Authors:** Nicholas Chadi, Emile Diamant, Tamara Perez, Afnan Al-Saleh, Marie-Pierre Sylvestre, Jennifer O’Loughlin, Jonathan P Winickoff, Olivier Drouin

**Affiliations:** 1 Sainte-Justine University Hospital Research Centre Montreal, QC, QC Canada; 2 Division of General Pediatrics, Department of Pediatrics Sainte-Justine University Hospital Centre Montréal, QC Canada; 3 Department of Pediatrics Université de Montréal Montréal, QC Canada; 4 Department of Medicine Université de Montréal Montreal, QC Canada; 5 Centre de recherche du centre hospitalier de l’Université de Montréal Montreal, QC Canada; 6 Department of Social and Preventive Medicine School of Public Health Université de Montréal Montreal, QC Canada; 7 Department of Pediatrics Massachusetts General Hospital Harvard University Boston, MA United States

**Keywords:** adolescents, cessation, e-cigarettes, parents, pediatric clinic, screening, tobacco

## Abstract

**Background:**

Though rates of tobacco smoking have decreased consistently over the past 3 decades, cigarette use remains the top preventable cause of premature death in North America. The Clinical Effort Against Secondhand Smoke Exposure (CEASE) is a medical clinic-based intervention that systematically screens parents for tobacco use and offers them direct access to evidence-based smoking cessation services. While the effectiveness of CEASE for parents who smoke has already been demonstrated in the United States, the CEASE model has not yet been tested in Canada, among parents who use e-cigarettes, or among adolescents who use cigarettes and e-cigarettes.

**Objective:**

We aim to demonstrate the feasibility and evaluate the preliminary effectiveness of the CEASE program for parental smoking cessation and its adapted version for adolescent smoking cessation and adolescent and parental vaping cessation.

**Methods:**

We will approach parents or guardians of children aged between 0 and 17 years, as well as adolescent patients aged between 14 and 17 years, from a tertiary care pediatric hospital in Montreal, Quebec, Canada, for participation in this single-blinded, pilot randomized controlled trial. Eligible participants are those who report using tobacco cigarettes or e-cigarettes at least once in the last 7 days and present to an outpatient pediatric clinic for a scheduled appointment. Our recruitment target is 100 participants: 50 parents or guardians of children aged 17 years or younger, and 50 adolescents aged between 14 and 17 years. The feasibility of implementation of the CEASE model will be measured by recruitment and retention rates for all 4 participant groups (stratified as follows: parents who use cigarettes, parents who use e-cigarettes exclusively, adolescents who use cigarettes, and adolescents who use e-cigarettes exclusively). Parent and adolescent participants within each group are randomized to the intervention and control groups using a 1:1 ratio through a computer-generated randomization list. Preliminary effectiveness outcomes include self-reported smoking and e-cigarette cessation, use of cessation resources, changes in smoking and e-cigarette use, motivation to quit, and quit attempts among participants. Participants complete electronic questionnaires on a tablet in the clinic at baseline as well as electronic follow-up questionnaires at 1, 3, and 6 months. Individuals reporting successful quit attempts are invited to provide a urine sample for cotinine testing to biochemically confirm quit. Analyses include descriptive statistics as well as exploratory trajectory analyses of smoking, e-cigarette use, and motivation to quit.

**Results:**

Research activities began in June 2022. Participant enrollment and data collection began in February 2023 and are expected to be completed in 15 months.

**Conclusions:**

There is a strong need for effective and cost-effective smoking and vaping cessation interventions for parents and adolescents. If successful, this study will help inform the preparation of a fully powered randomized controlled trial of CEASE in Canada in these populations.

**Trial Registration:**

Clinicaltrials.gov NCT05366790; https://www.clinicaltrials.gov/study/NCT05366790

**International Registered Report Identifier (IRRID):**

DERR1-10.2196/47978

## Introduction

Tobacco use is the single greatest preventable cause of morbidity and mortality in Canada, accounting for 48,000 deaths and CAD $16.2 billion (US $11.9 billion) in attributable health-related costs annually [1,2]. Smoking is a significant risk factor for multiple cancers [3] as well as cardiovascular diseases [4], thus shortening the life expectancy of smokers who initiate during adolescence by more than 10 years [4]. Despite parental smoking being an important risk factor for child initiation of tobacco (children of parents who smoke have 4-fold greater odds of becoming smokers when compared with children of nonsmoking parents) as well as for the development of several illnesses, such as bronchiolitis, asthma exacerbations, and pneumonia [5], about 15% of Canadian children are still exposed to household secondhand smoke [6]. Parents are thus an important target for smoking cessation interventions to improve their own health and protect that of their children and future generations. Given that 90% of smokers start smoking before the age of 18 years [7], adolescence represents a critical period to intervene in the fight against smoking [8].

Smoking among adolescents and young adults remains common despite rates decreasing over the past 3 decades, and adolescent use of nicotine-containing e-cigarettes (hereafter referred to as e-cigarettes) has shown a worrying increase over the past 10 years [9]. Most adults who use e-cigarettes (e-cigarette use is referred to interchangeably with vaping) do not have a history of tobacco use; however, once vaping has been initiated, the risk of subsequent tobacco, alcohol, and drug use increases [9]. Commonly used e-cigarettes usually contain high concentrations of nicotine, and while potentially effective for adult smoking cessation [10], they are not effective for youth smoking cessation and should not be recommended in this population [11].

In 2019, more than half of smokers in Canada (57.9%) reported seriously considering quitting in the next 6 months, and 26.9% were considering doing so in the next month [12]. Similarly, the majority of adults and adolescents who use e-cigarettes report that they intend to quit vaping [13,14]. Individuals trying to quit without behavioral support or smoking cessation medication (eg, nicotine replacement therapy [NRT]) can face great challenges due to nicotine’s addictive nature and severe psychological and physical withdrawal symptoms [3]. Several evidence-based smoking cessation interventions exist, including behavioral and pharmacological treatments that can be used alone or in combination [15], which greatly increase the odds of successfully quitting [16], and even more so when behavioral and pharmacological (NRT) interventions are combined [17]. Behavioral support traditionally refers to counseling; however, newer modalities, such as SMS text messages and app-based interventions, have shown promise [16] and would provide individuals with personalized, accessible behavioral support in quitting at low to no cost.

Health care providers can enhance the success of quit attempts by offering assistance and facilitating access to smoking cessation interventions [18]. Through their regular contact with families, pediatric health care providers are in a unique position to assist parents and adolescents in quitting smoking and vaping. However, studies and clinical experience show that pediatric health care providers rarely capitalize on this opportunity [19]. Lack of time, lower perceived priority of smoking or e-cigarette cessation relative to other care, fear of upsetting families, and lack of perceived efficacy are among the reasons why this type of intervention is rarely provided [20]. Digital preappointment screening questionnaires to identify individuals who smoke or vape and connect them with existing resources are a promising avenue to deliver cessation interventions in pediatric clinics [21]. This approach is well-accepted by patients and health care providers [21].

The Clinical Effort Against Secondhand Smoke Exposure (CEASE) intervention is an effective and cost-effective medical clinic-based intervention that systematically screens parents for tobacco use and offers them direct access to evidence-based smoking cessation services [22,23]. The model has been studied in the United States with promising results. A cluster randomized trial showed a 3.7% decrease in smoking prevalence between the intervention and control groups over a 2-year observation period (2.7% decrease in smoking rates in the intervention group vs 1.1% increase in the control group; difference –3.7%, 95% CI –6.3% to –1.2%; [23]). Cost-effectiveness data from Drouin et al [22] also showed that the incremental cost per quit (among adult participants who smoked) was US $1132 compared with usual care, a much lower figure than other clinical care-based smoking cessation interventions.

The primary objectives of this pilot randomized controlled trial of CEASE are to demonstrate the feasibility and preliminary effectiveness of CEASE for adults who smoke tobacco and its adapted version for parents with e-cigarette use and adolescents with cigarette and e-cigarette use in Canada. This pilot trial seeks to accomplish the following three aims: (1) evaluate the feasibility and effectiveness of the CEASE model in the Canadian setting, which differs from the United States in pediatricians’ scope of practice, financial incentives for physicians, and insurance coverage for NRT [24]; (2) address the gaps in knowledge regarding vaping cessation, for which many professional societies recommend using similar cessation tactics, strategies, and resources as with regular tobacco cigarettes [11,25]; and (3) validate the feasibility of recruiting participants in proposed sites of care (hospital-based pediatric clinics) and obtaining data on quit interest and intent in this population.

This pilot randomized controlled trial of CEASE in Canada aims to answer these questions by recruiting parent and adolescent participants from hospital-based pediatric clinics and randomizing them to the CEASE program or usual care.

## Methods

### Study Design, Setting, and Participants

**Figure 1:**
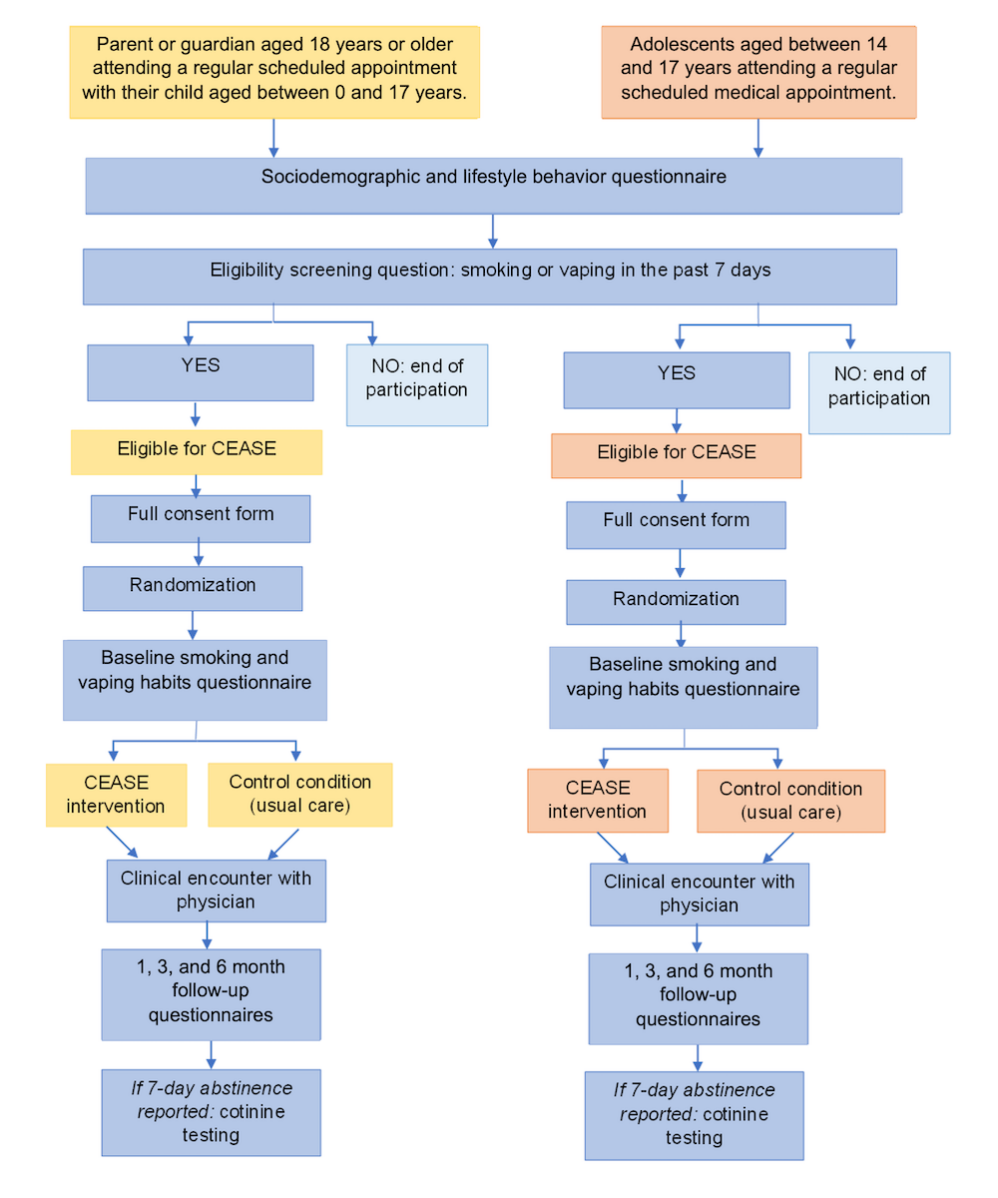
Study flow diagram illustrating participant trajectory through study of a brief digital screening and intervention tool for parental and adolescent tobacco and electronic cigarette use. CEASE: Clinical Effort Against Secondhand Smoke Exposure.

This is a single-center, pragmatic, single-blind, pilot randomized controlled trial comparing the CEASE intervention to usual care (control condition). Participants will be recruited over a 9-month period and followed for 6 months (15-month study period). Figure 1 shows the study flow diagram. This study is being conducted in various clinics within the Sainte-Justine University Hospital Centre, (Montreal, Quebec, Canada), including the general pediatrics clinic, the adolescent medicine clinic, and the orthopedics and sports medicine clinics.

The recruitment target is 100 individuals who report using tobacco or e-cigarettes (either tobacco or e-cigarettes alone, or both tobacco and e-cigarettes) in the 7 days before responding to the study questionnaire. A minimum of 50 parents and 50 adolescents will be recruited.

The study will be reported according to the Strengthening the Reporting of Observational Studies in Epidemiology Statement: Guidelines for Reporting Observational Studies [26].

### Recruitment

The appointment schedules of participating clinics will be reviewed the week before by the research team, and a recruitment schedule will be made according to the clinical volume of each clinic to maximize the number of potential patients approached. At the time of registration for their scheduled appointment, parents or legal guardians (hereafter referred to as parents) of children aged between 0 and 17 years as well as adolescents aged between 14 and 17 years are approached by a research assistant in the clinic waiting room to complete the screening survey on an electronic tablet. The survey is preceded by an abbreviated consent form. This approach is similar to that which was successfully used in CEASE studies in pediatric primary care in the United States [23]. Parents and adolescents are asked sociodemographic, smoking, and e-cigarette screening questions (see “Inclusion and Exclusion Criteria” section). Participants who respond “yes” to at least 1 of the 2 screening questions are invited to participate in the CEASE pilot study and have access to the full consent form. These forms are available in the Multimedia Appendix 1. During the entire study period, research assistants keep a detailed recruitment log, including information on participant screening, eligibility, recruitment, randomization, and retention in the study.

### Inclusion and Exclusion Criteria

#### Inclusion Criteria

Parents of children aged between 0 and 17 years who smoke cigarettes or use nicotine-containing e-cigarettes. Users are defined as those who answer “yes” to at least 1 of the 2 screening questions: (1) Have you smoked a single cigarette, even a puff, in the past 7 days? (smoking), and (2) Have you used an e-cigarette or vaping device containing nicotine, even a puff, in the past 7 days? (vaping).

Parents are eligible if (1) they are aged 18 years or older, (2) their child is aged between 0 and 17 years, (3) they are attending a regular scheduled medical appointment, and (4) they are sufficiently proficient in either French or English to be able to read and answer a written questionnaire. Informed consent is obtained before data collection.

Adolescent users are defined as those who answer “yes” to at least 1 of the above screening questions.

Adolescents are eligible if they are aged between 14 and 17 years, meet the aforementioned 3rd and 4th criteria, and have provided informed consent (in Québec, adolescents aged 14 years can provide consent).

#### Exclusion Criteria

Adolescents whose parents (if present) are not agreeable to their participation and families presenting to the clinic for an urgent medical issue will be excluded.

In each age category, a total of 2 participant groups are formed: “smokers,” defined as all users reporting past 7-day use of traditional combustible cigarettes, with or without e-cigarette use, and “e-cigarette-only users,” who report exclusive use of e-cigarettes. E-cigarettes include any electronic nicotine delivery systems, for example, vape pens, e-pens, e-cigars, e-pipes, or vaporizers containing nicotine. This division was created on the basis that the CEASE model has only been tested in individuals who report cigarette use (with or without e-cigarette use) but has not yet been tested in individuals using exclusively e-cigarettes [22].

### Intervention

#### The CEASE Intervention

The CEASE intervention is an evidence-based parental smoking cessation intervention delivered in pediatric practices, leveraging existing health care and community resources [23]. In this project, CEASE is delivered through electronic tablets in pediatric clinic waiting rooms. It integrates evidence-informed tobacco (cigarette) and vaping (e-cigarette) product use screening and cessation assistance into routine visits to pediatric clinics. This workflow, used by Winickoff et al [19] in the United States, requires less than 10 minutes to complete, does not disrupt clinic flow, can be sustained over a 2-year period, and is acceptable to families and health care providers. CEASE is based on the 5As (ask about smoking, advise to quit, assess readiness to quit, assist with a quit plan, and arrange follow-up) model of smoking cessation. Given that CEASE is a 1-time intervention, “arrange” is removed, and the fourth step “assist” is divided into 2 parts: (1) providing behavioral support (in-person, phone, SMS text message, or app quit support) and (2) providing pharmacological support (access to NRT). Specifically, CEASE includes the following steps:

1. Ask (routine, systematic identification of smokers and e-cigarette users)
2. Advise (brief motivational intervention)
3. Assess (self-assessment of readiness and quitting preferences)
4. Assist (support to quit for those interested)
  - Behavioral support
  - Pharmacological support (NRT)

#### Ask: Routine, Systematic Identification of Smokers and E-Cigarette Users

Families with a scheduled appointment are approached by a research assistant and prescreened for eligibility using a brief anonymous survey on the secure REDCap (Research Electronic Data Capture; Vanderbilt University) platform. Eligible participants are randomized to either (1) the intervention (CEASE) or (2) the control (usual care condition).

#### Advise: Brief Motivational Intervention

Brief motivational interventions have been shown to improve quit rates and may include goal setting, setting a quit date, secondhand smoke education (shown to increase acceptance of services), and enlisting social support. After collecting data on participant smoking and vaping behaviors (a baseline questionnaire delivered through an electronic tablet), CEASE will automatically include brief motivational messages describing the main short- and long-term health benefits of smoking cessation to increase participant engagement and acceptance of services [23].

#### Assess: Self-Assessment of Readiness and Quitting Preferences

Next, participants are invited to indicate whether they are interested in trying behavioral (eg, in-person, phone, SMS text message, or app-based) or pharmacological (ie, NRT) interventions to help them quit or cut down.

#### Assist: Support to Quit for Those Interested—Behavioral Support

In-person or phone quit line support is offered through the government-funded and evidence-based smoking cessation internet platform “J’Arrête (I Quit Now),” provided by the Conseil Québécois sur le Tabac et la Santé (CQTS). Interested individuals can sign up for cessation services directly on the website [27]; study participants have the opportunity to do so directly on the electronic tablet. The Canadian Cancer Society’s (CCS) Short Messages Against Tobacco [28], available from the J’Arrête (I Quit Now) website, is also presented as an option for participants who are interested in receiving SMS text messaging support to help them quit. Participants are offered to sign up to the “J’Arrête (I Quit Now)” web-based app, which provides a platform for the creation and realization of their smoking quit plan, as well as a dashboard to track their progress, trophies, tricks, and a variety of exercises to help overcome smoking cravings. In the week following the completion of the baseline questionnaire, interested participants receive an email or SMS text message reminder with information on how to access the behavioral resources they selected. All in-person, web-based, and SMS text messaging services are available in French and English for both adults and adolescents who smoke and use e-cigarettes. After signifying their interest in signing up for either of these services, participants are asked to consent to their information being transferred to the CCS or to the CQTS, which may contact them in the future and offer them additional smoking or vaping cessation resources.

#### Assist: Support to Quit for Those Interested—Pharmacological Support

CEASE automatically generates a written recommendation for NRT [23], to be discussed with a community pharmacist, including for those cutting down to quit as recommended in the latest national and international guidelines [29]. Since the best practice for NRT is to provide both a long- (ie, patch) and short-acting form (eg, gums or lozenges), a recommendation for 2 forms of NRT is automatically populated and printed in the clinic, provided to the participant, and sent to the participant by email. Given that pharmacists can now prescribe NRT in Quebec, the recommendation is written as detailed instructions for the pharmacist to prescribe NRT for a period of 3 months (the time period covered by public medical insurance in Quebec) and adjust the dosage based on the participant’s reported use of tobacco or e-cigarettes. Study investigators remain available to support community pharmacists as needed.

The role of the provider in this intervention is adjunctive and not necessary to trigger intervention delivery. As such, CEASE is a standalone intervention and can take place without any provider involvement.

#### Control Condition

The control condition is care as is usually delivered in participating clinics. Current practice at those sites does not currently include routine provision of assistance for parental nor adolescent smoking and e-cigarette cessation (eg, referral to quit lines and NRT prescription). Participants in the control conditions will complete the same questionnaire as those in the intervention group; however, they will not be presented with a list of resources at the end of the questionnaire. While completing the final 6-month follow-up questionnaire, participants in the control group will be offered the option to receive a 1-time direct linkage to CEASE smoking and vaping cessation resources (behavioral supports and NRT recommendation).

### Data Collection and Compensation

After consenting to the study, participants are asked to fill out a baseline questionnaire on an electronic tablet. Participants randomized to the intervention group have the option of receiving direct access to the CEASE evidence-informed cessation resources (if interested) after completing their questionnaire, while those randomized to the control group are only offered access to these resources at the end of the 6-month follow-up period (see “Control Condition” section). Participants in both groups are followed for 6 months following recruitment, with data collection at 1, 3, and 6 months (and the possibility of obtaining direct access to evidence-informed resources at each time point, if interested, for participants in the intervention group). Data collection tools (questionnaires) are available upon reasonable request to the corresponding author. Participant confidentiality is protected by limiting the collection of identifying information to an email address and phone number; date of birth, name, or participant address will not be collected. All data are stored on the secure web-based REDCap platform and in study logs on the Sainte-Justine University Hospital Centre server without participant-identifying information. Only the study investigators will have access to this data set at the end of the study period.

At each time point, participants are contacted by the study team by email, phone, or SMS text message (based on their preference) with a notification to complete the follow-up survey through REDCap, a secure web-based platform. If participants declare an abstinence of 7 days or more and consent to undergoing urine cotinine testing, they are contacted to arrange for biochemical confirmation of smoking cessation (cotinine testing) [30]. Data for confirmatory smoking or vaping cessation testing (urinary cotinine testing results) is sent directly to the study coordinator (to maintain investigator blinding) using a secure web-based portal. Participants will be called by research assistants and sent reminder emails or SMS text messages (based on their preference) to bolster the completion of follow-up questionnaires. They will receive CAD $10 (US $7.31) in compensation for each survey completed and an additional CAD $20 (US $14.62) per urine sample provided for cotinine testing.

Recruitment is expected to last a period of 9 months, with an additional 6 months required for follow-up with all recruited participants to complete data collection. The data analysis will begin with the end of recruitment and is expected to be completed in the 9 months following the final participant follow-up, for a total of a 24-month study period.

### Allocation

This study aims to recruit an equal number of parent and adolescent users. Parent and adolescent participants are randomized to the intervention and control groups using a 1:1 ratio. Parent and adolescent participants are stratified into 2 subgroups: those who smoke traditional combustible cigarettes (with or without e-cigarette use) and those who report exclusive use of e-cigarettes. A computer-generated randomization list created by a biostatistician external to the study team is used to randomize participants within each group.

### Blinding

This is a single-blinded study in which investigators will be blinded to the participant’s group allocation, and data analysis is blinded, but participants and research assistants remain unblinded. Pediatricians may decide to discuss smoking and vaping with families as part of their usual care, and participants may mention the CEASE intervention during the encounter with the provider. As such, participants and physicians will not be blinded. Participant data is anonymized using individual participant codes. No participant identifiers (eg, birth date and hospital number) are included in the database.

### Outcomes

The capacity of CEASE to provide cessation assistance for parental and adolescent smoking and e-cigarette use is assessed using a series of outcomes detailed in Tables 1 and 2. Table 1 includes all clinic-level outcomes, while Table 2 includes all patient-level outcomes. Each table includes details and a summary of each outcome measure, their operational definition, as well as the suggested time line for data collection and feasibility threshold when applicable. Outcomes were based on Winickoff’s previous experience conducting CEASE feasibility and effectiveness trials in the United States [23].

Feasibility thresholds of 50% of potential participants screened for eligibility, 15% clinic-level smoking and vaping prevalence, 20% enrollment rate, and 3 participants (total) recruited per week (at least 6 parents and 6 adolescents recruited per month; primary outcome for this study) have been set (Table 1). Given an average of 250 patients seen per week across all 4 clinic recruitment sites, we anticipate that these thresholds will allow for 100 participants to be recruited over 36 weeks (9 months). Similarly, a feasibility threshold for baseline and follow-up questionnaire completion has been set at 90% and 80%, respectively, as shown in Table 2.

Data are obtained directly from participants through baseline and follow-up questionnaires, as well as from recruitment logs, biochemical assay results, and key informant interviews with clinic staff (eg, clinical nurse, front desk staff, and medical director) to inform about the potential impacts of the study on clinic flow. The key informant semistructured interview template is presented in Multimedia Appendix 2.

**Table 1:** Clinic-level outcomes of a pilot randomized controlled trial evaluating the feasibility and preliminary effectiveness in Canadian pediatric clinics of a brief digital screening and intervention tool for parental and adolescent tobacco and electronic cigarette use.

Outcome				Operational definition and details of measure	Timeline	Data collection	Feasibility threshold
**Recruitment**							
	Clinic flow			Number of potential participants (parent and adolescent) per month for each clinic	Throughout	Recruitment log	N/A^a^
	Proportion screened			Proportion of potential participants screened for eligibility	Throughout	Recruitment log	>50%
	Clinic-level smoking and vaping prevalence			Self-reported smoking and vaping rates among parents and adolescents screened	Throughout	Recruitment log	>15%
	Eligibility rate			Number of eligible parents and adolescents per month for each clinic	Throughout	Recruitment log	N/A
	Enrollment percentage			Proportion of eligible participants approached who are enrolled	Throughout	Recruitment log	>20%
	Reason for declining			Reasons for declining participation in the study	Throughout	Recruitment log	N/A
	Recruitment rate (primary outcome)			Total number of participants recruited per month (parents and adolescents)	Throughout	Recruitment log	>3 participants, total, per week; >6 adolescents and >6 parents per month
**Feasibility of the intervention**							
			Interest in smoking or vaping cessation	Proportion of eligible participants per category (parents and adolescents) who declare interest in smoking or vaping cessation	End of study	Baseline and follow-up questionnaires	>5%
			Interest in smoking or vaping cessation services	Proportion of eligible participants (parents and adolescents) who want to be connected with smoking or vaping cessation services (quit lines, etc)	End of study	Baseline and follow-up questionnaires	>5%
			Interest in obtaining NRT^b^	Proportion of eligible participants in each category who want to receive NRT	End of study	Baseline and follow-up questionnaires	>5%
**Impact on clinic workflow**							
		Clinic staff evaluation		Key informant semistructured interview template (Multimedia Appendix 2) based on a number of references, including the CFIR^c^ interview guide [31] and RE-AIM^d^ [32]	End of study	Key informant interviews	N/A
		Patient flow		Pre- and postrecruitment average weekly and monthly patient volumes in each clinic	End of study	Key informant interviews	<10% change

^a^N/A: not applicable.

^b^NRT: nicotine replacement therapy.

^c^CFIR: Consolidated Framework for Implementation Research.

^d^RE-AIM: Reach, Effectiveness, Adoption, Implementation, and Maintenance.

**Table 2:** Participant-level outcomes of a pilot randomized controlled trial evaluating the feasibility and preliminary effectiveness in Canadian pediatric clinics of a brief digital screening and intervention tool for parental and adolescent tobacco and electronic cigarette use.

Outcome		Operational definition and details of measure	Timeline				Data collection	Feasibility threshold
			Baseline	1 month	3 months	6 months		
**Protocol fidelity**								
	Baseline questionnaire completion	Proportion of participants who complete the baseline questionnaire out of randomized participants (REDCap^a^ survey)	✓				Baseline questionnaire	≥90%
	Access to web-based resources	Proportion of participants who want to be connected to the web-based resources who report access these resources (self-report)	✓	✓	✓	✓	Baseline and follow-up questionnaires	N/A^b^
	Access to quit lines (phone, or SMS text messaging support)	Proportion of interested participants who received a communication from the quit lines (self-report)		✓	✓	✓	Baseline and follow-up questionnaires	N/A
	Access to NRT^c^	Proportion of participants who want to receive NRT who receive a report receiving a formal written recommendation or prescription for NRT	✓	✓	✓	✓	Baseline and follow-up questionnaires	N/A
	NRT use	Proportion of participants who have used NRT (self-report)		✓	✓	✓	Baseline and follow-up questionnaires	N/A
**Study retention**								
	Participant retention	Proportion of randomized participants that could be contacted (initiation of REDCap survey, phone, or email contact) at follow-up		✓	✓	✓	Follow-up questionnaires	≥80%
	Smoking and vaping status	Availability of self-reported smoking and vaping status for randomized participants		✓	✓	✓	Follow-up questionnaires	≥80%
	Completion of data at follow-up	Complete survey data available for randomized participants		✓	✓	✓	Follow-up questionnaires	≥60%
	Cotinine testing	Availability of cotinine testing results		✓	✓	✓	Web-based portal	N/A

^a^REDCap: Research Electronic Data Capture.

^b^N/A: not applicable.

^c^NRT: nicotine replacement therapy.

### Power Calculations

The most recent Statistics Canada data show prevalence rates for daily or occasional smoking that are higher among males (17.3%) than females (12.3%), though this gap is narrowing [33]. In Québec, where the study is conducted, smoking prevalence is slightly higher (17.0%) than the Canadian average (14.8%) [33]. Among adults, especially adults aged older than 25 years, smoking rates remain significantly higher than vaping rates, as only 3.2% of adults aged older than 25 years report past-month use of a vaping device, and a significant proportion of adult e-cigarette users report dual use of cigarettes and vaping devices [34].

The latest provincial statistics report an 18% vaping prevalence among youth aged between 15 and 17 years [35] in Quebec, which is 4.5 times higher than the smoking prevalence (4%) in this age group, though the majority of adolescents who smoke also report past use of an electronic cigarette [34].

Considering recent epidemiological trends, a conservative 15% prevalence estimate is expected for the combined use of cigarettes and vaping devices among both parents and adolescents, resulting in a potential participant pool of approximately 1 in 7 parents or adolescents presenting to the clinic. With average clinic volumes of 250 patients per week and the screening and enrollment rates mentioned above, we anticipate attaining targeted recruitment rates of 3 participants per week.

To estimate the retention rate, using a sample size of 50 participants per age group (parents and adolescents) will allow for a 95% CI within 15% of the estimate obtained. As such, if a study completion rate of 60% is observed, the 95% CI would be 44.2%-75.8%. Other research groups have reported challenges in recruiting teenagers for vaping cessation research studies [36]. As such, recruitment and retention rates are expected to differ between adult and adolescent participants; enrolling 50 parents and 50 adolescents for this pilot study will provide the 2 participation rate estimates.

### Analyses

Clinic-level outcomes will be evaluated using descriptive statistics (recruitment and feasibility) and qualitatively (key informant interviews). Participant-level outcomes will also be evaluated using descriptive statistics, both overall and stratified by participant type (parents vs adolescents), nicotine product used (cigarettes with or without vaping vs vaping only), and clinic where recruitment occurred. Recruitment outcomes will be compared by participant gender. Given that this is a pilot trial with insufficient power to formally test the effectiveness of CEASE, exploratory analyses comparing smoking and vaping cessation, use of cessation resources, and motivation to quit smoking and vaping between intervention and control participants will be performed to identify potential trends and signals of effectiveness in all 4 participant groups.

### Ethical Considerations

This study was reviewed and approved by the Institutional Review Board of the Sainte-Justine University Hospital Research Centre (2023-4237) and has been registered on ClinicalTrials.gov (NCT05366790). Any modifications or deviations from the protocol or adverse events will be recorded in the study logs on REDCap and reported immediately (within 48 hours) to the Institutional Review Board of the Sainte-Justine University Hospital Research Centre.

## Results

This study obtained funding in April 2022. Research activities for this study began in June 2022. Participant enrollment and data collection began in February 2023 and are expected to be completed in 9 months (October 2023). As of September 30, 2023, a total of 115 participants—61 parents and 54 adolescents—have been recruited and have completed all baseline data. Data analysis will begin once all participants have been recruited and follow-up measures (1, 3, and 6-month follow-up) are in progress. The primary results of this study are expected to be available in the first months of 2025.

## Discussion

### Potential

This is the first study to test the feasibility and preliminary effectiveness of the CEASE intervention in Canada, as well as with adolescents and individuals with exclusive e-cigarette use. If shown feasible by reaching the feasibility recruitment threshold of 6 parent and 6 adolescent participants per month, this study could allow for the planning of a full-scale randomized controlled trial testing CEASE’s effectiveness in Canada in these populations.

### Strengths and Limitations

This study includes several strengths. First, it builds on the CEASE intervention, which has previously been demonstrated to be effective and cost-effective. Second, the electronic format of the screening and intervention will facilitate the integration of the tool in clinic workflow without any required intervention from clinical staff and will help with scale-up to additional sites. Finally, the study leverages existing cigarette and e-cigarette cessation resources, foregoing the need for additional costs for resource development. This also means that those resources can evolve and improve over time without affecting the design or feasibility of the study.

This study has certain limitations. Given the pilot nature of the study and the small sample size, this study does not have the statistical power to evaluate the effectiveness of the CEASE intervention. This study is also being conducted exclusively in an urban tertiary care hospital, which may not be reflective of pediatric clinics in community hospitals or other primary care clinics. Nonetheless, as recruitment is taking place in both general pediatric and adolescent medicine clinics as well as several specialized pediatric clinics, it allows for testing of the CEASE model in a diverse parent and adolescent patient group. Given this diversity of participants and recruitment clinics within a single center, we elected to randomize at the participant level rather than following the cluster-randomization protocol previously used to evaluate CEASE [23]. This decision does, however, pose a risk of contamination, wherein participants in the control group learn of the intervention and choose to seek out these publicly available smoking cessation resources themselves. While this may result in a decreased effect size of “The Clinical Effort Against Secondhand Smoke Exposure–Canada” (CanCEASE) in this study, all participants are offered access to these resources at the end of the study period, and the CanCEASE questionnaire itself may prompt individuals wishing to quit smoking or vaping to seek out these publicly available resources themselves during the study. Furthermore, cluster randomization would have been impractical in the context of this pilot study, as a larger sample size is required.

Logical next steps for our work will be to consider expanding CEASE recruitment to other clinic settings, including primary and secondary care clinics as well as rural settings, and conducting a full-scale cluster randomized controlled trial.

### Conclusions

Cigarette smoking and nicotine vaping remain highly prevalent among parents and adolescents. Pediatric clinics represent an underutilized opportunity for the promotion of healthy lifestyle behaviors. This study tests the feasibility and preliminary effectiveness of a simple approach to smoking and vaping cessation. If successful, it could pave the way for a large-scale randomized controlled trial testing the effectiveness of this approach for adolescents and parents who smoke and vape in a Canadian setting. It also has the potential to inform clinicians and researchers about preferred cessation tools for parents and adolescents who use cigarettes and e-cigarettes, which remains an understudied area.

It is anticipated, based on previous implementation of CEASE in the United States, that CEASE will be feasible, as determined by a satisfactory recruitment rate as well as high proportions of participants interested in cessation, referral to cessation services, and NRT. As such, the data obtained through this study will help inform the design and implementation of a fully powered randomized controlled trial of CEASE in Canada.

## Appendix

Multimedia Appendix 1 Consent form (English) for participation in a randomized-controlled pilot trial evaluating the feasibility and preliminary effectiveness in Canadian pediatric clinics of a brief digital screening and intervention tool for parental and adolescent tobacco and electronic cigarette use.

Multimedia Appendix 2 Key informant interviews.

Multimedia Appendix 3 Peer Review Reports from the Canadian Institutes of Health Research (CIHR).

## Abbreviations

CanCEASE: The Clinical Effort Against Secondhand Smoke Exposure–Canada
CCS: Canadian Cancer Society
CEASE: Clinical Effort Against Secondhand Smoke Exposure
CQTS: Conseil Québécois sur le Tabac et la Santé
NRT: nicotine replacement therapy
REDCap: Research Electronic Data Capture

## Data Availability

The data sets generated during and/or analyzed during this study are available from the corresponding author on reasonable request.
