# Supplementary material for: A Brief Digital Screening and Intervention Tool for Parental and Adolescent Tobacco and Electronic Cigarette Use in Pediatric Medical Care in Canada: Protocol for a Pilot Randomized Controlled Trial
Source: JMIR Res Protoc. 2023 Nov 30;12:e47978. doi: 10.2196/47978 (PMC10722363; doi:10.2196/47978)
Supplement: Multimedia Appendix 1 [file resprot_v12i1e47978_app1.docx]

**
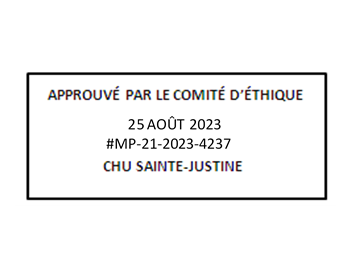
**
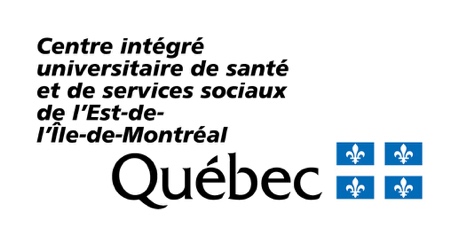

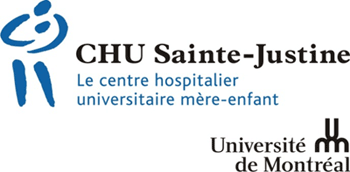


**RESEARCH INFORMATION AND CONSENT FORM**

**Title:** **Nicotine cessation intervention for parents and adolescents in pediatric medical care: feasibility study of the CanCEASE program**

**Persons responsible:**

Olivier Drouin, MD, MSc, MPH, Division of General Paediatrics, Department of Pediatrics, CHU Sainte-Justine

Nicholas Chadi, MD, MPH, Department of Paediatrics, CHU Sainte-Justine

Noémie Desjardins, MD, Department of Paediatrics, Hôpital Maisonneuve-Rosemont

**Other researchers:**

- Université de Montréal, School of Public Health: Jennifer O’Loughlin, PhD; Marie-Pierre Sylvestre, PhD
- Massachusetts General Hospital & Harvard Medical School: Jonathan P. Winickoff, MD, MPH, FAAP

**Funding Source:** Canadian Institutes of Health Research

**WHY ARE YOU BEING INVITED TO TAKE PART IN THIS STUDY?**

Tobacco use and nicotine vaping carry several risks for human health. Smoking and vaping can also have harmful effects on family members, especially children who may be at risk of negative health effects due to exposure to secondhand smoke.

Today, we are inviting you to take part in a research project in which we are testing a brief smoking/vaping cessation intervention delivered on this tablet. If you use tobacco or vaping products and are either a parent/guardian of a child aged 0-18 years or a teen aged 14-17 years-old attending a scheduled clinic appointment at the CHU Sainte-Justine or Hôpital Maisonneuve Rosemont, you will be eligible for this study. The people who receive the intervention will be determined at random, based on an algorithm programmed in the tablet. Please read the following information to help you decide if you want to participate in this research study. It is important that you understand this information. We encourage you to ask questions. Please take all the time you need to make your decision.

**WHY IS THIS STUDY BEING DONE?**

Several studies have shown that short in-person or digital interventions (for example, using tablets) can help adults and adolescents quit smoking. In this study, we are testing an intervention called CEASE, which was first designed in the United States to help parents quit smoking. This is the first study testing the CEASE program in Canada. The CEASE program is also being tested to support smoking and vaping cessation with adolescents in a pediatric clinic. If successful, this program could be brought to other clinics across the country to help parents and adolescents quit smoking and vaping.

**HOW MANY PEOPLE WILL TAKE PART IN THIS STUDY?**

Approximately 140 participants; 70 parents and 70 adolescents, will take part in this study.

**WHAT WILL HAPPEN DURING THIS RESEARCH STUDY?**

Your participation in the project will include the following:

1. Completion of a questionnaire of approximately 10 minutes about your smoking and vaping habits and your interest and readiness to change these habits.
2. You will be placed at random in a group that will either receive direct access to smoking/vaping cessation resources throughout the 6-month study period or care as usual, with access to these resources at the end of the study.
3. Regardless of which group you will be placed in, you will receive a short electronic questionnaire, sent by text message or email, to complete after 1, 3, and 6 months.
4. At the end of the study (after 6 months), if you were in the groups that didn’t receive access to smoking/vaping cessation resources, you will have the option to receive them then.

If you report a successful quit attempt at 1,3, or 6 months, you will be invited to provide a urine sample which will help confirm that there is no more nicotine in your system. Detailed instructions on how to provide this urine sample will be provided. To compensate you for your time, a 20$ compensation will be given to you if you submit the sample. You will also be given 10$ for completing the questionnaire at 1-month and 20$ for completing both of the remaining questionnaires at 3 and 6 months.

**FOR HOW LONG WILL YOU PARTICIPATE IN THIS STUDY?**

Participants in this study will be followed for a period of 6 months. This follow-up period includes the questionnaires we will send you 1, 3, and 6-months after you enter the study. We may also contact you after this study is over to participate in related future studies.

**ARE THERE BENEFITS TO TAKING PART IN THE STUDY?**

Your participation in this study will provide you access to evidence-based resources to help you quit smoking/vaping. By participating in this study, you will be contributing to the advancement of knowledge to improve smoking cessation interventions.

**WHAT ARE THE RISKS?**

The risks of this study are minimal. There is no inconvenience other than the time it takes to answer the questionnaires and provide urine samples (if it applies to you). You may choose not to respond to certain questions or to end your participation at any time. A research assistant will be available to respond to any of your questions while you complete the first questionnaire and throughout the duration of the study period.

**HOW WILL THE INFORMATION PROVIDED BE KEPT CONFIDENTIAL?**

All the information obtained during the study will be kept confidential as required or permitted by law. Your identity will be protected on all documents by replacing your name with a research number. Only the research team will have access to the code linking your name to this number.

In order to ensure your protection and quality control of the research project, representatives from the Research ethics committee of the CHU Sainte-Justine may consult your research records. They adhere to a confidentiality policy.

If the general results of this study are published or presented at scientific meetings, your name and other personal information will not be used.

The principal investigators will be responsible for storing all research data for 7 years on a secure server.

**IS YOUR PARTICIPATION VOLUNTARY?**

Yes. Taking part in this study is voluntary. You may choose not to be in this study. You can decide to stop being in the study at any time. If you decide not to be in this study, or to stop participating in the study later on, this will not affect the quality of care received at the clinic. Should you choose to stop participating, the data already collected from you will be destroyed, however those that have already been analyzed will be stored for the integrity of the research results.

**WHO DO I CALL IF I HAVE QUESTIONS OR PROBLEMS?**

If you have any questions about this research project, you can contact the researchers or research study coordinator responsible for the project in your hospital:

Dr. Olivier Drouin at (514) 345-4931, extension 4226 or contact him via email at [olivier.drouin.hsj@ssss.gouv.qc.ca](mailto:olivier.drouin.hsj@ssss.gouv.qc.ca)

Dr. Nicholas Chadi at 514-345-4931 ext. 4101 or contact him via email at [nicholas.chadi.med@ssss.gouv.qc.ca](mailto:nicholas.chadi.med@ssss.gouv.qc.ca)

Study coordinator: Tamara Perez, MSc at [tamara.perez.hsj@ssss.gouv.qc.ca](mailto:tamara.perez.hsj@ssss.gouv.qc.ca)

If you would like information about your rights related to your participation in the research, you may contact the hospital Ombudsperson (Patient Representative):

- CHU Sainte-Justine: (514) 345-4749
- Hôpital Maisonneuve-Rosemont: [commissaireauxplaintes.cemtl@ssss.gouv.qc.ca](mailto:commissaireauxplaintes.cemtl@ssss.gouv.qc.ca) or (514) 252-3510.

**RESEARCH ETHICS COMMITTEE**

The research ethics committee of the CHU Sainte Justine and of the CIUSSS de l’Est-de-l’Île-de-Montréal approved this project and will monitor the project.

**CONSENT**

**Project Title: Nicotine cessation intervention for parents and adolescents in pediatric medical care: feasibility study of the CanCEASE program**

I have been explained what will happen during this study. I read the information and consent form and will be sent an electronic copy to keep. I was able to ask my questions and they were answered to my satisfaction. After thinking about it, I agree to participate in this research project.

In no way does consenting to participate in this research study waive your legal rights nor release the sponsor or the institution from their legal or professional responsibilities if you are harmed in any way.

🞏 I agree to participate in this study

Name of participant (parent or adolescent) Signature Date

(Type) (Use finger)

I have explained to the participant all the relevant aspects of this study. I answered any questions they asked. I explained that participation in a research project is free and voluntary and that they are free to stop participating at any time they choose.

Name of Person obtaining consent (signature) Date

🞏 I prefer not to participate in this study

If participant agrees to participate:

Thank you for accepting to take part in this study!

Contact information and communication preferences:

Your answers to the following questions will be used to contact you for the purposes of this study.

Please type your phone number in the space provided. _______________

How would you prefer to be contacted for this study (to receive a copy of the consent form and electronic link for follow-up questionnaires)?

🞏 Email

🞏 Text message

🞏 Email and text message

What is time of day is best for you to be contacted regarding this research project?

- Morning
- Afternoon
- Evening
- Any time

In which language would you prefer to be contacted for this study?

- French
- English

**If participant is NOT interested in participating in the study**

Would you please indicate the reason(s) you are not interested in participating in the study?

- I’ve tried to quit in the past and it didn’t work
- I don’t want to quit smoking/vaping
- I’m not interested in talking about this topic
- I don’t have the time today
- I don’t want to participate in a research study
- Other: ___________

🡺You may return the tablet to the research assistant.

Thank you for your time and have a good day!
