# Supplementary material for: A Brief Digital Screening and Intervention Tool for Parental and Adolescent Tobacco and Electronic Cigarette Use in Pediatric Medical Care in Canada: Protocol for a Pilot Randomized Controlled Trial
Source: JMIR Res Protoc. 2023 Nov 30;12:e47978. doi: 10.2196/47978 (PMC10722363; doi:10.2196/47978)
Supplement: Multimedia Appendix 2 [file resprot_v12i1e47978_app2.docx]

Appendix 1: Key informant semi-structured interview template

1. What is your role at the clinic (ex. doctor, nurse, administrative staff…)?
2. How long have you been working at the clinic?
3. Did you feel that the fact that your clinic participated in this research project required you to perform additional work?
   1. If so, can you briefly explain what this work consisted of?
   2. Can you also provide an estimate of the added workload, per week, on average (ex. how many minutes)?
4. Did you feel that this project required you to adjust your daily clinic tasks?
5. If so, how did it impact your clinical workflow?
6. Did you find ways to integrate project tasks into your workflow?
7. What kinds of questions did families ask about this project?
8. Did you feel that you were able to adequately respond to their questions?
9. If you contacted researchers responsible or research assistants for this project for help answering questions, were they able to help you respond in a way that was satisfactory and timely? If no, please elaborate.
10. Were you faced with any pushback or negative feedback from families?
11. If so, please describe these situations and the negative feedback expressed.
12. Did you feel that you were able to respond to pushback from families?
13. Did families offer any suggestions regarding the project?
14. If so, please describe any suggestions received from families regarding this project.
15. Do you have any suggestions to improve this project’s workflow and integration into your clinical workflow?
16. Do you have any suggestions to make this smoking/vaping cessation intervention more effective?
17. Do you feel that this project had an impact on your relationship and/or clinical rapport with families?
18. If so, please describe the nature of the impact.
19. Is there any additional information you would like to be provided with regarding smoking & vaping cessation?
    1. Are there any additional resources you would like to see developed regarding smoking & vaping cessation?
20. Since the implementation of this project, would you say that you discussed tobacco use or vaping with families during clinic visits more than before, less than before, or about the same?
21. (FOR CLINICAL PROVIDERS ONLY) Were you able to guess which parents or patients were participating in the study?
22. (FOR CLINICAL PROVIDERS ONLY) Were you able to guess which arm of the study (intervention vs. control) parents or patients participating in the study were in?
23. (FOR CLINICAL PROVIDERS ONLY) How often do you document parental tobacco use or vaping in the clinic?
24. For patients 14-17 years, how often do you document smoking/vaping in the clinic note?
25. Has this project increased/decreased/no change how often you document parental or teen smoking and vaping in your clinical notes?
26. How do you feel about the integration of smoking/vaping cessation for parents and teens in the waiting rooms of pediatric clinics?
27. Do you have any questions or suggestions regarding this study?
