# Supplementary material for: A Brief Digital Screening and Intervention Tool for Parental and Adolescent Tobacco and Electronic Cigarette Use in Pediatric Medical Care in Canada: Protocol for a Pilot Randomized Controlled Trial
Source: JMIR Res Protoc. 2023 Nov 30;12:e47978. doi: 10.2196/47978 (PMC10722363; doi:10.2196/47978)
Supplement: Multimedia Appendix 3 [file resprot_v12i1e47978_app3.pdf]

Canadian Institutes of Health Research
160 Elgin Street, 9th Floor
Address Locator 4809A
Ottawa, Ontario K1A 0W9

Instituts de recherche en santé du Canada
160, rue Elgin, 9e étage
Indice de l'adresse 4809A
Ottawa (Ontario) K1A 0W9

Le 2 février 2022

Institute of Aging
Institute of Cancer Research
Institute of Circulatory and Respiratory Health
Institute of Gender and Health
Institute of Genetics
Institute of Health Services and Policy Research
Institute of Human Development and Child and Youth Health
Institute of Indigenous Peoples' Health
Institute of Infection and Immunity
Institute of Musculoskeletal Health and Arthritis
Institute of Neurosciences, Mental Health and Addiction
Institute of Nutrition, Metabolism and Diabetes
Institute of Population and Public Health

Institut du vieillissement
Institut du cancer
Institut de la santé circulatoire et respiratoire
Institut de la santé des femmes et des hommes
Institut de génétique
Institut des services et des politiques de la santé
Institut du développement et de la santé des enfants et des adolescents
Institut de la santé des Autochtones
Institut des maladies infectieuses et immunitaires
Institut de l'appareil locomoteur et de l'arthrite
Institut des neurosciences, de la santé mentale et des toxicomanies
Institut de la nutrition, du métabolisme et du diabète
Institut de la santé publique et des populations

Docteur Olivier Drouin
CHU Sainte-Justine
Service de pédiatrie générale
3175 Chemin de la Côte-Sainte-Catherine
7e étage, Bloc 9
Montreal, Québec H3T 1C5

Docteur Drouin,

Les Instituts de recherche en santé du Canada (IRSC) ont évalué votre demande intitulée « Application of a brief digital screening tool to address parental and adolescent tobacco and electronic cigarette use in pediatric medical care », présentée au concours de subventions Projet de l'automne 2021. Malheureusement, elle n'a pas été retenue pour le budget et la durée du financement demandés.

Toutefois, j'ai le plaisir de vous informer que vous recevrez un financement spécifique par l'entremise de l'annonce de priorités Équité en matière de prévention du cancer et de lutte contre cette maladie. Le financement de ce projet est fourni par l'Institut du cancer (IC) des IRSC.

Les évaluations et les résultats de votre demande sont accessibles dans RechercheNet. Si vous ne pouvez accéder aux documents, veuillez communiquer avec nous à support-soutien@cihr-irsc.gc.ca. Votre autorisation de financement vous sera envoyée par courriel.

Il convient de souligner que le nombre de demandes approuvées (indiqué dans l'avis de décision) comprend les demandes figurant au-dessus du seuil de financement établi pour le comité et, le cas échéant, les demandes financées dans le cadre du processus de rééquilibrage tel que décrit dans Le processus décisionnel du Programme de subventions Projet sur notre site Web (https://cihr-irsc.gc.ca/f/52317.html). Le processus de rééquilibrage est en place afin que la proportion de subventions financées au sein des cohortes admissibles au rééquilibrage soit au moins égale à celle des demandes soumises par les cohortes admissibles au concours.

Veuillez noter que vous devez mentionner l'apport financier de l'annonce de priorités indiquée ci-dessus dans toutes les communications et publications liées à ce projet.

Étant donné que les IRSC n'informent pas les cocandidats de leur décision, nous vous prions de communiquer le résultat de cette demande aux personnes concernées et à leurs établissements de recherche (s'ils diffèrent du vôtre).

Pour toute question, n'hésitez pas à communiquer avec un agent de traitement du centre de contact par téléphone au 613-954-1968 ou par courriel à support-soutien@cihr-irsc.gc.ca.

Sincères salutations,

Paula Kirton

Paula Kirton
Manager, Program Design and Delivery
Research Programs Portfolio

Canada

CIHR IRSC
Discoveries for life / Découvertes pour la vie

Review Type/Type d'évaluation: SO Notes /Notes de l'agent scientifique
Name of Applicant/Nom du chercheur: Drouin, Olivier
Application No./Numéro de demande: 470909
Agency/Agence: CIHR/IRSC
Competition/Concours: 2021-09-15 Project Grant/Subvention Projet
Committee/Comité: Health Services Evaluation & Interventions Research 3/Recherche en interventions et en évaluation dans les services de santé 3
Title/Titre: Application of a brief digital screening tool to address parental and adolescent tobacco and electronic cigarette use in pediatric medical care

---

Assessment/Évaluation:

Strengths (including SGBA considerations):

The intervention is interesting and novel, and the question is important.
There is significant potential impact on health of youth and young adults.
The research team is very strong with experience in relevant areas, and leverages existing resources.
Knowledge users are engaged in the study, and KT activities are sound and well described.

Weaknesses (including SGBA considerations):

The committee questioned the rationale for an RCT. What is the justification for an RCT/effectiveness study as opposed to an implementation study or quasi-experimental evaluation, in light of existing RCT evidence in the US supporting the effectiveness of the intervention? The intervention appears to be effective based on previous research. There may be differences between the US and Canada, but it is not clear why this should be evaluated using an RCT design.
The study will primarily occur in acute care settings rather than community settings; this may not be reflective of current practice.
The committee had concerns about who had accountability for the NRT prescriptions if they are automatically generated.
More detail about how electronic messages will be customized and delivered would be helpful, as would additional detail about what CEASE+ is.
The study questions could be written more clearly (eg PICO framework).
Pilot data regarding the CEASE intervention would strengthen the feasibility of the application. Further, why are pilot data needed for CEASE+ but not for CEASE? What is the justification for sample size of 40 in the pilot study?
Analysis of sex and gender were not incorporated into the protocol.

Budget:

****

Note: The final rating of the application, provided in the Notice of Recommendation (NOR) and Notice of Decision (NOD), is the averaged rating of the peer review committee members following the discussion of the application during the committee meeting, and therefore may differ from the ratings provided by the assigned reviewers in their respective reviews.

Remarque : La cote définitive de la demande, qui apparaît dans l’avis de recommandation et l’avis de décision, représente la moyenne des cotes accordées par les membres du comité d’évaluation par les pairs après avoir débattu de la demande à la réunion du comité. Elle peut donc différer de celle donnée par les évaluateurs dans leur évaluation respective.
``````````````````````````````````````````````````````````````````````````````````````````````````````````````````````````
``````````````````````````````````````````````````````````````````````````````````````````````````````````````````````````

**Review Type / Type d'évaluation:**	Reviewer 1 / Évaluateur 1
**Name of Applicant / Nom du chercheur:**	Drouin, Olivier
**Application No. / Numéro de demande:**	470909
**Agency / Agence:**	CIHR/IRSC
**Competition / Concours:**	Project Grant/Subvention Projet
**Committee / Comité:**	Health Services Evaluation & Interventions Research 3/Recherche en interventions et en évaluation dans les services de santé 3
**Title / Titre:**	Application of a brief digital screening tool to address parental and adolescent tobacco and electronic cigarette use in pediatric medical care

Adjudication Criteria/Critères de sélection

Initial Score/Cote Initiale: 4.0

Top/Bottom Selection/Groupe supérieur/inférieur

- (◉) **Top/Groupe supérieur**
- (○) **Bottom/Groupe inférieur**

**Review Type / Type d'évaluation:**	Reviewer 1 / Évaluateur 1
**Name of Applicant / Nom du chercheur:**	Drouin, Olivier
**Application No. / Numéro de demande:**	470909
**Agency / Agence:**	CIHR/IRSC
**Competition / Concours:**	Project Grant/Subvention Projet
**Committee / Comité:**	Health Services Evaluation & Interventions Research 3/Recherche en interventions et en évaluation dans les services de santé 3
**Title / Titre:**	Application of a brief digital screening tool to address parental and adolescent tobacco and electronic cigarette use in pediatric medical care

Summary of Application/Résumé de la demande:

The proposed study aims to assess CEASE (Clinical Effort Against Secondhand Smoke) in Canada. CEASE has been demonstrated as effective and cost-effective in the US. CEASE uses the 5As approach in pediatric settings to promote smoking cessation of parents. An ancillary pilot study of the feasibility of a trial of CEASE+ is also proposed to help parents quit vaping and youth quit both smoking and vaping. 460 smoking parents will be randomized to usual care or CEASE in four hospital-based pediatric clinics. Proposed is a multicentric, pragmatic randomized-control trial allocated to either CEASE or usual care in four highly diverse hospital-based pediatric clinics (including lower SES and racial/ethnic minority groups) in Montréal, Québec. CEASE includes systematic pre-appointment screening for parental smoking in clinic waiting rooms using electronic tablets and provides direct linkage with existing smoking/vaping cessation resources including publicly funded quitlines, text message and app-based services and/or NRT. Outcomes are 7 day point prevalence at 6 months post-intervention.

**Review Type / Type d'évaluation:**	Reviewer 1 / Évaluateur 1
**Name of Applicant / Nom du chercheur:**	Drouin, Olivier
**Application No. / Numéro de demande:**	470909
**Agency / Agence:**	CIHR/IRSC
**Competition / Concours:**	Project Grant/Subvention Projet
**Committee / Comité:**	Health Services Evaluation & Interventions Research 3/Recherche en interventions et en évaluation dans les services de santé 3
**Title / Titre:**	Application of a brief digital screening tool to address parental and adolescent tobacco and electronic cigarette use in pediatric medical care

Strengths and Weaknesses/Forces et faiblesses:

Strengths: excellent team, tremendous experience, well designed.

Weakness: Main purpose is to assess whether CEASE which had been demonstrated to be cost-effective in US studies is effective and cost-effective in Canada.

At present, CEASE has only been tested in the U.S., and evaluated only using clinic-level

randomization. Important differences exist in pediatric preventive care delivery between the U.S. and Canada, including pediatricians' scope of practice, financial incentives for physicians, and insurance coverage for NRT. 57 These could influence the effectiveness of CEASE, warranting its formal evaluation in Canada. Our team proposes the first RCT of CEASE in Canada, in hospital-based pediatric clinics and using participant-level randomization. We also seek to explore the feasibility of using CEASE to help parents quit vaping and adolescents quit smoking and vaping, avenues which have not yet been tested.

However, the trial does not test the actual practices in Canada, but rather a funded study in which physicians don't actually generally provide any treatment and in which NRT prescriptions are automatically generated. Moreover, the proposed trial would take place only in acute care settings and not in regular community pediatric practices where most pediatric care is provided. It is therefore not clear that the proposed trial would be testing typical Canadian settings that would be substantially different from American ones.

**Review Type / Type d'évaluation:**	Reviewer 1 / Évaluateur 1
**Name of Applicant / Nom du chercheur:**	Drouin, Olivier
**Application No. / Numéro de demande:**	470909
**Agency / Agence:**	CIHR/IRSC
**Competition / Concours:**	Project Grant/Subvention Projet
**Committee / Comité:**	Health Services Evaluation & Interventions Research 3/Recherche en interventions et en évaluation dans les services de santé 3
**Title / Titre:**	Application of a brief digital screening tool to address parental and adolescent tobacco and electronic cigarette use in pediatric medical care

Budget Recommendation/Recommandation budgétaire:

4 full-time RAs – one for each site for 1.5 years in addition to Coordinator at 21 hours per week and PhD student for 3 years. Each site will have only about 115 participants. Full-time RA for 18 months seems a bit much. (Total budget is $550k)

Please indicate your appraisal of the integration of sex as a biological variable as a strength, weakness, or not applicable to the proposal./Prière de sélectionner une option pour donner votre évaluation de l'intégration du sexe comme variable biologique en tant que point fort ou point faible de la proposition, ou en tant qu'élément non applicable à la proposition.

- (●) **Strength/Point fort**
- ( ) **Weakness/Point faible**
- ( ) **Not applicable/Non applicable**

Please indicate your appraisal of the integration of gender as a socio-cultural determinant of health as a strength, weakness, or not applicable to the proposal./Prière de sélectionner une option pour donner votre évaluation de l'intégration du genre comme déterminant socioculturel de la santé en tant que point fort ou point faible de la proposition, ou en tant qu'élément non applicable à la proposition.

- (●) **Strength/Point fort**
- ( ) **Weakness/Point faible**
- ( ) **Not applicable/Non applicable**

**Review Type / Type d'évaluation:**	Reviewer 1 / Évaluateur 1
**Name of Applicant / Nom du chercheur:**	Drouin, Olivier
**Application No. / Numéro de demande:**	470909
**Agency / Agence:**	CIHR/IRSC
**Competition / Concours:**	Project Grant/Subvention Projet
**Committee / Comité:**	Health Services Evaluation & Interventions Research 3/Recherche en interventions et en évaluation dans les services de santé 3
**Title / Titre:**	Application of a brief digital screening tool to address parental and adolescent tobacco and electronic cigarette use in pediatric medical care

Sex and/or Gender Considerations/Notions de sexe et/ou de genre:

not mentioned in the proposal itself except to say that females have lower rates of smoking and are more likely to participate. The SGBA section does specify that both sex and gender data will be collected and addressed in the analysis and that recruitment of staff will consider sex/gender ratios. One of the investigators is an expert.

**Review Type / Type d'évaluation:**	Reviewer 2 / Évaluateur 2
**Name of Applicant / Nom du chercheur:**	Drouin, Olivier
**Application No. / Numéro de demande:**	470909
**Agency / Agence:**	CIHR/IRSC
**Competition / Concours:**	Project Grant/Subvention Projet
**Committee / Comité:**	Health Services Evaluation & Interventions Research 3/Recherche en interventions et en évaluation dans les services de santé 3
**Title / Titre:**	Application of a brief digital screening tool to address parental and adolescent tobacco and electronic cigarette use in pediatric medical care

Adjudication Criteria/Critères de sélection

Initial Score/Cote Initiale: 3.7

Top/Bottom Selection/Groupe supérieur/inférieur

○ **Top/Groupe supérieur**
◉ **Bottom/Groupe inférieur**

**Review Type / Type d'évaluation:**	Reviewer 2 / Évaluateur 2
**Name of Applicant / Nom du chercheur:**	Drouin, Olivier
**Application No. / Numéro de demande:**	470909
**Agency / Agence:**	CIHR/IRSC
**Competition / Concours:**	Project Grant/Subvention Projet
**Committee / Comité:**	Health Services Evaluation & Interventions Research 3/Recherche en interventions et en évaluation dans les services de santé 3
**Title / Titre:**	Application of a brief digital screening tool to address parental and adolescent tobacco and electronic cigarette use in pediatric medical care

Summary of Application/Résumé de la demande:

The aim of the proposed research is to evaluate the effectiveness of the Clinical Effort Against Secondhand Smoke (CEASE) - an evidence-based parental smoking cessation intervention that screens parents for tobacco use during their child’s pediatrician appointment and refers them to cessation services. The feasibility of an adapted version of CEASE (CEASE +) to help parents quit vaping and adolescents quit smoking and vaping will also be tested.

The research team will conduct a multi-centre, pragmatic, randomized control trial of parents who smoke. A total of 460 parents will be recruited from 4 highly diverse hospital-based pediatric clinics in Montreal and randomized to either CEASE/CEASE + or usual care, after pre-appointment screening for smoking. Individuals randomized to CEASE/CEASE+ will have motivational messages delivered to them via a study tablet. Participants are then invited to indicate if they want to try behavioral or pharmacological interventions to help them quit or reduce smoking. Quitline support via phone or text message will be offered. CEASE/CEASE+ will automatically generate a prescription for nicotine replacement therapy. The primary study outcomes will be parent-reported quit at 6 months (with 3 month quit also being measured). Secondary outcome measures will include meaningful assistance (i.e., referral to services or nicotine-replacement therapy) in quitting smoking, and cost-effectiveness.

The team envisions positive results from this trial could warrant use of the intervention across Canada. This, in turn, would have an impact on decreasing adult and adolescent smoking rates, and have positive implications for tobacco-related morbidity and mortality.

Strengths and Weaknesses/Forces et faiblesses:

A.Significance and Impact of the Research

Strengths

The research team makes a strong argument as to the impact of tobacco use on individual morbidity and mortality, including risk of cancer and cardiovascular disease. The team also effectively argues about the impact on parental smoking on not only the parent themselves but also the child (and its implications for the health care system).

The intervention (CEASE) is an interesting (and potentially innovative) approach to target parental smoking via their child's pediatric health care appointment.

The proposed intervention – if feasible and effective - could have a positive impact on both the short-term and long-term health of youth and adults.

Weaknesses

Despite the importance of a family-centred care approach in the pediatric setting, might one argue that "treating" parents within the context of pediatric care is "beyond the scope" of what should be done in a pediatric setting especially when medical health care professionals are licensed to provide care to a specific population. What is the acceptability of this type of approach by pediatricians, for example, beyond the context of the study?

One of the arguments as to the significance of the problem is that tobacco screening and support happens less than 5% of the time in pediatric practices. Although the researchers' point is understandable, it may be viewed as not a strong argument as the immediate goal of the pediatric health care professional would be to intervene with the child (and not the parent) unless the presenting problem was directly related to actions of the parent (e.g., asthma exacerbation and parenteral smoking in the home). Although it is foreseeable that family-centred care would include the parent, more exploration of the point presented may be important.

The primary goal of the research is clearly delineated. However, the addition of specific research questions might be helpful. The hypothesis related to the effectiveness of the CEASE intervention is a 5% higher quit rate in those in the intervention versus the control. The support for 5% and why this is a "big enough" change is not substantiated.

The CEASE intervention was developed and tested in the United States. Although the research team recognizes factors that may influence its effectiveness in a Canadian context which is an argument for testing the intervention in Canada, more details about the initial CEASE study to understand whether it is a robust intervention would strengthen the research team's argument for this intervention.

B.Approaches and Methods

Strengths

RCT design to study the effectiveness of the CEASE/CEASE +

Randomization to the arms of the study occurs with a central system and will be stratified.

Intervention is based on the 5A model of smoking cessation which is a strength (the "last "a" however, has been modified as there will be no follow-up).

Plan to utilize quit resources that currently exist within the system.

Sample size and power calculation conducted. Research team has considered who will be available for the study – recruitment rate based on current census of people. Unclear if this rate is impacted by current COVID-19 restrictions.

Already secured collaboration from 4 study sites with letters of support.

Objectively measuring the outcome of quitting via a urine sample (cotinine-confirmed quit) is good. How will you determine if the participant actually sent in their sample and not that of a non-smoking family member?

Included an examination of cost-effectiveness, although more detail would be helpful.

Weaknesses

The study leverages local healthcare and community resources which is good. But, how are these "programs" integrated into the intervention?

Part of the intervention is motivational messages to the individual being screened via the tablet. How these messages are generated and then delivered to the tablet is not clear.

In the "assess" phase, participants will be invited to indicate whether they want to try a behavioural intervention to help them quit. How will the research team ensure the intervention is actually being delivered the same way to participants since, for example, quit support by phone would be quite different than text-messaging).

Exactly what is CEASE + - it is not really clear. Further elaboration is needed.

As part of the intervention, there is an automatic generation of a prescription. What about the legalities surrounding this? How is the “dose” determined? Would this be considered a pediatrician generating a prescription for the adult since they are at a pediatric appointment (even though the prescription is automatic)? Would it be considered outside physician’s “scope of practice”? Who is accountable?

Although objectively measuring the outcome of quitting via a urine sample (cotinine-confirmed quit) is a strength, how will you determine if the participant actually sent in their urine sample and not that of a non-smoking family member?

Ethics – concerns over control group not receiving any information/resources. Ethically, would a clinician ignore that a client needs help and not provide them with any support? Is there another strategy that might be used?

Are there strategies to control for co-intervention or contamination? For example, people in the control group – during the study period – might choose to access support services.

Suggestion: The research team might consider a higher level of compensation for participants depending on the length of time to complete the questionnaire.

C.Expertise, Experience and Resources
The research team appears to the have the skills and expertise to successfully conduct the study. The team is comprised of a mix of new/early, mid and senior investigators. Collectively, they have expertise in pediatrics, public health, tobacco control and addiction, health services research, and biostatistics. The Nominated Principal Applicant is an early career researcher and clinician-scientist; they have training in general pediatrics and graduate studies in public health, as well as completed a research fellowship in pediatric health services. They hold a 4-year Clinical Research Scholar 1 Salary Award (Quebec), and a CIHR Project Grant on asthma medication adherence and outcomes, as well as being a Co-Investigator on an international project funded by CIHR. The Co-PA is a clinician-scientist in adolescent and addiction medicine and has conducted a cost-effectiveness analysis of the CEASE program in the US. Collaborators from each of the study sites, and knowledge users from the Canadian Cancer Society, Quebec Tobacco and Health, and the Centre for Health Promotion at CHUSJ complement the team.

The inclusion of a trainee to the team would be an excellent opportunity for mentorship and trainee learning.

**Review Type / Type d'évaluation:**	Reviewer 2 / Évaluateur 2
**Name of Applicant / Nom du chercheur:**	Drouin, Olivier
**Application No. / Numéro de demande:**	470909
**Agency / Agence:**	CIHR/IRSC
**Competition / Concours:**	Project Grant/Subvention Projet
**Committee / Comité:**	Health Services Evaluation & Interventions Research 3/Recherche en interventions et en évaluation dans les services de santé 3
**Title / Titre:**	Application of a brief digital screening tool to address parental and adolescent tobacco and electronic cigarette use in pediatric medical care

Budget Recommendation/Recommandation budgétaire:

No recommended change to the budget. The NPA currently holds CIHR funding but identified that there is no budgetary overlap with this proposed study.

**Review Type / Type d'évaluation:**	Reviewer 2 / Évaluateur 2
**Name of Applicant / Nom du chercheur:**	Drouin, Olivier
**Application No. / Numéro de demande:**	470909
**Agency / Agence:**	CIHR/IRSC
**Competition / Concours:**	Project Grant/Subvention Projet
**Committee / Comité:**	Health Services Evaluation & Interventions Research 3/Recherche en interventions et en évaluation dans les services de santé 3
**Title / Titre:**	Application of a brief digital screening tool to address parental and adolescent tobacco and electronic cigarette use in pediatric medical care

Please indicate your appraisal of the integration of sex as a biological variable as a strength, weakness, or not applicable to the proposal./Prière de sélectionner une option pour donner votre évaluation de l'intégration du sexe comme variable biologique en tant que point fort ou point faible de la proposition, ou en tant qu'élément non applicable à la proposition.

- ◉ **Strength/Point fort**
- ○ **Weakness/Point faible**
- ○ **Not applicable/Non applicable**

Please indicate your appraisal of the integration of gender as a socio-cultural determinant of health as a strength, weakness, or not applicable to the proposal./Prière de sélectionner une option pour donner votre évaluation de l'intégration du genre comme déterminant socioculturel de la santé en tant que point fort ou point faible de la proposition, ou en tant qu'élément non applicable à la proposition.

- ◉ **Strength/Point fort**
- ○ **Weakness/Point faible**
- ○ **Not applicable/Non applicable**

**Review Type / Type d'évaluation:**	Reviewer 2 / Évaluateur 2
**Name of Applicant / Nom du chercheur:**	Drouin, Olivier
**Application No. / Numéro de demande:**	470909
**Agency / Agence:**	CIHR/IRSC
**Competition / Concours:**	Project Grant/Subvention Projet
**Committee / Comité:**	Health Services Evaluation & Interventions Research 3/Recherche en interventions et en évaluation dans les services de santé 3
**Title / Titre:**	Application of a brief digital screening tool to address parental and adolescent tobacco and electronic cigarette use in pediatric medical care

Sex and/or Gender Considerations/Notions de sexe et/ou de genre:

Sex as a biological variable

• Sex and gender will be collected from participants.

• Researchers identified smoking rates are higher in males, and transgender and non-binary individuals than individuals who identify as cisgender; plan to actively recruit male participants since the CEASE intervention in the US yielded 4:1 female: male ratio.

Gender as a socio-cultural determinant of health

• Stratified gender-base analysis will be conducted.

• Gender will be used in pilot exploratory analyses for e-cigarette cessation.

• In addition to these considerations, team members will conduct online training on sex- and gender-based analysis if not yet completed.

• The researchers note that one of the team members is a director of a youth gender identity clinic and part of a research team on transgender youth and families. Another team member has published on sex and gender differences in smoking in youth.

**Review Type / Type d'évaluation:**	Reviewer 3 / Évaluateur 3
**Name of Applicant / Nom du chercheur:**	Drouin, Olivier
**Application No. / Numéro de demande:**	470909
**Agency / Agence:**	CIHR/IRSC
**Competition / Concours:**	Project Grant/Subvention Projet
**Committee / Comité:**	Health Services Evaluation & Interventions Research 3/Recherche en interventions et en évaluation dans les services de santé 3
**Title / Titre:**	Application of a brief digital screening tool to address parental and adolescent tobacco and electronic cigarette use in pediatric medical care

Adjudication Criteria/Critères de sélection

Initial Score/Cote Initiale: 3.8

Top/Bottom Selection/Groupe supérieur/inférieur

- ○ **Top/Groupe supérieur**
- ◉ **Bottom/Groupe inférieur**

**Review Type / Type d'évaluation:**	Reviewer 3 / Évaluateur 3
**Name of Applicant / Nom du chercheur:**	Drouin, Olivier
**Application No. / Numéro de demande:**	470909
**Agency / Agence:**	CIHR/IRSC
**Competition / Concours:**	Project Grant/Subvention Projet
**Committee / Comité:**	Health Services Evaluation & Interventions Research 3/Recherche en interventions et en évaluation dans les services de santé 3
**Title / Titre:**	Application of a brief digital screening tool to address parental and adolescent tobacco and electronic cigarette use in pediatric medical care

Summary of Application/Résumé de la demande:

The applicants propose a multicenter, pragmatic, randomized-control trial aimed to evaluate the effectiveness of a parental smoking cessation intervention, the Clinical Effort Against Second-hand Smoke (CEASE), in a Canadian setting. The CEASE is an intervention developed in the U.S. to systematically screen parents for tobacco use and refer them to evidence-based cessation services. Secondarily, the applicants aim to conduct an ancillary pilot study on the feasibility of using an adapted version of CEASE (CEASE+) to help parents quit vaping and to assist adolescents in quitting smoking and vaping.

In the main RCT trial, parents who smoke will be recruited in four hospital-based pediatric clinics in Montréal, Québec, and allocated to either CEASE or usual care. CEASE includes systematic pre-appointment screening for parental smoking in clinic waiting rooms using electronic tablets and provides direct linkage with existing smoking/vaping cessation resources. These resources include publicly funded quit lines, text message and app-based services and/or nicotine-replacement therapy. The primary outcome of interest is parent-reported quitting at 6 months post-intervention. Parent-reported quitting at 3 months will be an interim outcome. Secondary outcomes will include proportion of parents who received meaningful assistance in quitting smoking (referral to behavioural services or nicotine-replacement therapy) as well as cost-effectiveness of the intervention. The targeted sample size for the main trial is 460 participants (230 per group) based on an a priori sample size calculation (targeting a 5.5% increase in the percentage of smoking parents who quit). Primary analysis will be conducted using chi-square test to compare quitting rates in the intervention vs. control arm.

For the ancillary pilot study (CEASE+), parents who vape and adolescents who smoke or vape will be recruited. The applicants anticipate the recruitment of 40 participants. Feasibility outcomes will be reported descriptively and include recruitment rate, retention rate, data completeness, and impact on clinic flow.

**Review Type / Type d'évaluation:**	Reviewer 3 / Évaluateur 3
**Name of Applicant / Nom du chercheur:**	Drouin, Olivier
**Application No. / Numéro de demande:**	470909
**Agency / Agence:**	CIHR/IRSC
**Competition / Concours:**	Project Grant/Subvention Projet
**Committee / Comité:**	Health Services Evaluation & Interventions Research 3/Recherche en interventions et en évaluation dans les services de santé 3
**Title / Titre:**	Application of a brief digital screening tool to address parental and adolescent tobacco and electronic cigarette use in pediatric medical care

Strengths and Weaknesses/Forces et faiblesses:

Strengths:

•The study rationale is nicely written and provides a comprehensive overview of the literature on the study topic.

•The principal applicants have expertise and experience in the content area and in conducting randomized controlled trials.

•The proposal is supported by a comprehensive team of researchers and clinicians with complimentary experience and expertise. Their roles are described in the supplementary material

•The Knowledge Translation (KT) activities proposed are sound and clearly described.

•Sex and gender were thoughtfully taken into account in the research design, methods, and analysis plan.

•The application includes relevant letters of support, attesting for stakeholders' engagement.

•The timeline and related deliverables of the project seem realistic.

Weaknesses:

•The proposal would have been strengthened if the study aim/research question had been framed according to the PICO framework (https://bestpractice.bmj.com/info/toolkit/learn-ebm/how-to-clarify-a-clinical-question/).

•Compliance with reporting guidelines for trial protocols (https://www.spirit-statement.org) would have strengthened the proposal and ensured optimal description of trial methods.

•As described in the application, evidence from a cluster RCT conducted in the US supports the efficacy of the CEASE interventions in promoting smoking cessation among parents in pediatric settings. While the applicants suggest that differences in pediatric care delivery (i.e., including pediatricians' scope of practice, financial incentives for physicians, and insurance coverage for nicotine replacement therapy) warrants further evaluation of this intervention in Canada, I wonder if this evaluation requires a new comparative-effectiveness RCT. As, according to the applicants, the CEASE intervention is already deemed an 'evidence-based intervention', perhaps the next research steps to translate this knowledge to Canadian pediatric settings should focus on 'implementation research' rather than 'effectiveness research' (https://www.ncbi.nlm.nih.gov/pmc/articles/PMC7348037/). For example, quasi-experimental/observational studies may contribute important new evidence regarding real-world rates of adoption, reach, acceptability, fidelity, cost, and sustainability of this intervention in Canada. In my opinion, the need for a (complex and resource intensive) RCT to assess the comparative-effectiveness of CEASE in Canada requires further justification.

•If a Canada-based RCT is deemed necessary, given the above-mentioned differences in pediatric care delivery compared to the US, further information is required to support the feasibility of conducting such RCT in a Canadian healthcare setting. Due to the complexity inherent to well-designed RCTs, pilot/feasibility studies are a critical first step to test logistical needs, optimize the design, and build the capacities required for a full-scale trial. The lack of pilot data supporting the feasibility of the trial proposed is a limitation of this application.

•In line with the point above, the submission of RCT proposals to CIHR requires the use of specific headings. While the applicants partially complied to this requirement, heading 2.19 (‘Has any pilot study been carried out using this design?’) has been omitted from the application.

•I was a bit confused by the description of the blinding procedures proposed. According to the applicants, investigators will be blinded to the participants’ group allocation; however, many of these investigators are (not blinded) pedicatricians involved in the care of study participants (or their children). Perhaps the applicants meant that outcomes assessors will be blinded? If so, this is also debatable since all the study outcomes will be self-reported by unblinded patients.

•Since healthcare providers will not be blinded to treatment allocation, risk of performance bias (pediatricians providing further smoke cessation advise to patients randomized to CEASE or CEASE+) is an important concern that should be further addressed by the applicants.

•The proposal includes an ancillary pilot trial aimed to assess the feasibility of using an adapted version of CEASE (CEASE+) to help parents quit vaping, and to assist adolescents quit smoking and vaping. It is unclear how the intervention will be adapted to support other modes of nicotine delivery (e-cigarettes) and populations (adolescents and adults).
•It is unclear why a pilot study was deemed necessary to support the feasibility of a full-scale RCT focused on CEASE+. For CEASE intervention, the applicants proposed a full-scale RCT despite the lack of context-specific feasibility data. The rationale behind this decision requires further clarification. I would argue that feasibility trails are required before embarking in full-scale RCTs testing both interventions.

•The applicants estimate that approximately 40 participants will be recruited for the pilot trial, but it is unclear whether this sample size is sufficient to provide precise estimates of feasibility outcomes (including recruitment and retention rates, data completeness, and impact on clinic flow). The targeted sample size for the pilot trial proposed requires further justification (https://www.bmj.com/content/355/bmj.i5239).

•It is unclear how the feasibility outcome ‘impact on clinic flow’ will be quantified.

•The applicants set a feasibility threshold of >50% for the proportion of eligible participants approached. This seems to be a very low threshold – e.g., the approach of only 51% of the eligible participants may limit the generalizability of findings. Since recruitment will be done at clinic waiting rooms, perhaps expecting an approach rate closer to 100% would be

reasonable?

•The involvement of (a) patient-partner(s) would have strengthened this application by ensuring that patients' perspectives are taken into account in the study design and conduct.

**Review Type / Type d'évaluation:**	Reviewer 3 / Évaluateur 3
**Name of Applicant / Nom du chercheur:**	Drouin, Olivier
**Application No. / Numéro de demande:**	470909
**Agency / Agence:**	CIHR/IRSC
**Competition / Concours:**	Project Grant/Subvention Projet
**Committee / Comité:**	Health Services Evaluation & Interventions Research 3/Recherche en interventions et en évaluation dans les services de santé 3
**Title / Titre:**	Application of a brief digital screening tool to address parental and adolescent tobacco and electronic cigarette use in pediatric medical care

Budget Recommendation/Recommandation budgétaire:

The project budget seems appropriate.

**Review Type / Type d'évaluation:**	Reviewer 3 / Évaluateur 3
**Name of Applicant / Nom du chercheur:**	Drouin, Olivier
**Application No. / Numéro de demande:**	470909
**Agency / Agence:**	CIHR/IRSC
**Competition / Concours:**	Project Grant/Subvention Projet
**Committee / Comité:**	Health Services Evaluation & Interventions Research 3/Recherche en interventions et en évaluation dans les services de santé 3
**Title / Titre:**	Application of a brief digital screening tool to address parental and adolescent tobacco and electronic cigarette use in pediatric medical care

Please indicate your appraisal of the integration of sex as a biological variable as a strength, weakness, or not applicable to the proposal./Prière de sélectionner une option pour donner votre évaluation de l'intégration du sexe comme variable biologique en tant que point fort ou point faible de la proposition, ou en tant qu'élément non applicable à la proposition.

- (●) **Strength/Point fort**
- ( ) **Weakness/Point faible**
- ( ) **Not applicable/Non applicable**

Please indicate your appraisal of the integration of gender as a socio-cultural determinant of health as a strength, weakness, or not applicable to the proposal./Prière de sélectionner une option pour donner votre évaluation de l'intégration du genre comme déterminant socioculturel de la santé en tant que point fort ou point faible de la proposition, ou en tant qu'élément non applicable à la proposition.

- (●) **Strength/Point fort**
- ( ) **Weakness/Point faible**
- ( ) **Not applicable/Non applicable**

**Review Type / Type d'évaluation:**	Reviewer 3 / Évaluateur 3
**Name of Applicant / Nom du chercheur:**	Drouin, Olivier
**Application No. / Numéro de demande:**	470909
**Agency / Agence:**	CIHR/IRSC
**Competition / Concours:**	Project Grant/Subvention Projet
**Committee / Comité:**	Health Services Evaluation & Interventions Research 3/Recherche en interventions et en évaluation dans les services de santé 3
**Title / Titre:**	Application of a brief digital screening tool to address parental and adolescent tobacco and electronic cigarette use in pediatric medical care

Sex and/or Gender Considerations/Notions de sexe et/ou de genre:

•Sex and gender will be considered in every step of the study, including trial recruitment and analysis.

•All investigators will complete online training on sex- and gender-based analysis.

•When possible, research assistants will be hired using a 1:1 female to male ratio and positions will be actively promoted within sexual minority student and young professional groups.

•Some investigators are experienced with research focused on gender identity, sex and gender-related differences in the course of smoking.
